# Social determinants of health influence disease activity and functional disability in Polyarticular Juvenile Idiopathic Arthritis

**DOI:** 10.1186/s12969-022-00676-9

**Published:** 2022-03-07

**Authors:** William Daniel Soulsby, Nayimisha Balmuri, Victoria Cooley, Linda M. Gerber, Erica Lawson, Susan Goodman, Karen Onel, Bella Mehta, for the CARRA Registry Investigators, N. Abel, K. Abulaban, A. Adams, M. Adams, R. Agbayani, J. Aiello, S. Akoghlanian, C. Alejandro, E. Allenspach, R. Alperin, M. Alpizar, G. Amarilyo, W. Ambler, E. Anderson, S. Ardoin, S. Armendariz, E. Baker, I. Balboni, S. Balevic, L. Ballenger, S. Ballinger, N. Balmuri, F. Barbar-Smiley, L. Barillas-Arias, M. Basiaga, K. Baszis, M. Becker, H. Bell-Brunson, E. Beltz, H. Benham, S. Benseler, W. Bernal, T. Beukelman, T. Bigley, B. Binstadt, C. Black, M. Blakley, J. Bohnsack, J. Boland, A. Boneparth, S. Bowman, C. Bracaglia, E. Brooks, M. Brothers, A. Brown, H. Brunner, M. Buckley, M. Buckley, H. Bukulmez, D. Bullock, B. Cameron, S. Canna, L. Cannon, P. Carper, V. Cartwright, E. Cassidy, L. Cerracchio, E. Chalom, J. Chang, A. Chang-Hoftman, V. Chauhan, P. Chira, T. Chinn, K. Chundru, H. Clairman, D. Co, A. Confair, H. Conlon, R. Connor, A. Cooper, J. Cooper, S. Cooper, C. Correll, R. Corvalan, D. Costanzo, R. Cron, L. Curiel-Duran, T. Curington, M. Curry, A. Dalrymple, A. Davis, C. Davis, C. Davis, T. Davis, F. De Benedetti, D. De Ranieri, J. Dean, F. Dedeoglu, M. DeGuzman, N. Delnay, V. Dempsey, E. DeSantis, T. Dickson, J. Dingle, B. Donaldson, E. Dorsey, S. Dover, J. Dowling, J. Drew, K. Driest, Q. Du, K. Duarte, D. Durkee, E. Duverger, J. Dvergsten, A. Eberhard, M. Eckert, K. Ede, B. Edelheit, C. Edens, C. Edens, Y. Edgerly, M. Elder, B. Ervin, S. Fadrhonc, C. Failing, D. Fair, M. Falcon, L. Favier, S. Federici, B. Feldman, J. Fennell, I. Ferguson, P. Ferguson, B. Ferreira, R. Ferrucho, K. Fields, T. Finkel, M. Fitzgerald, C. Fleming, O. Flynn, L. Fogel, E. Fox, M. Fox, L. Franco, M. Freeman, K. Fritz, S. Froese, R. Fuhlbrigge, J. Fuller, N. George, K. Gerhold, D. Gerstbacher, M. Gilbert, M. Gillispie-Taylor, E. Giverc, C. Godiwala, I. Goh, H. Goheer, D. Goldsmith, E. Gotschlich, A. Gotte, B. Gottlieb, C. Gracia, T. Graham, S. Grevich, T. Griffin, J. Griswold, A. Grom, M. Guevara, P. Guittar, M. Guzman, M. Hager, T. Hahn, O. Halyabar, E. Hammelev, M. Hance, A. Hanson, L. Harel, S. Haro, J. Harris, O. Harry, E. Hartigan, J. Hausmann, A. Hay, K. Hayward, J. Heiart, K. Hekl, L. Henderson, M. Henrickson, A. Hersh, K. Hickey, P. Hill, S. Hillyer, L. Hiraki, M. Hiskey, P. Hobday, C. Hoffart, M. Holland, M. Hollander, S. Hong, M. Horwitz, J. Hsu, A. Huber, J. Huggins, J. Hui-Yuen, C. Hung, J. Huntington, A. Huttenlocher, M. Ibarra, L. Imundo, C. Inman, A. Insalaco, A. Jackson, S. Jackson, K. James, G. Janow, J. Jaquith, S. Jared, N. Johnson, J. Jones, J. Jones, J. Jones, K. Jones, S. Jones, S. Joshi, L. Jung, C. Justice, A. Justiniano, N. Karan, K. Kaufman, A. Kemp, E. Kessler, U. Khalsa, B. Kienzle, S. Kim, Y. Kimura, D. Kingsbury, M. Kitcharoensakkul, T. Klausmeier, K. Klein, M. Klein-Gitelman, B. Kompelien, A. Kosikowski, L. Kovalick, J. Kracker, S. Kramer, C. Kremer, J. Lai, J. Lam, B. Lang, S. Lapidus, B. Lapin, A. Lasky, D. Latham, E. Lawson, R. Laxer, P. Lee, P. Lee, T. Lee, L. Lentini, M. Lerman, D. Levy, S. Li, S. Lieberman, L. Lim, C. Lin, N. Ling, M. Lingis, M. Lo, D. Lovell, D. Lowman, N. Luca, S. Lvovich, C. Madison, J. Madison, S. Magni Manzoni, B. Malla, J. Maller, M. Malloy, M. Mannion, C. Manos, L. Marques, A. Martyniuk, T. Mason, S. Mathus, L. McAllister, K. McCarthy, K. McConnell, E. McCormick, D. McCurdy, P. Mc Curdy Stokes, S. McGuire, I. McHale, A. McMonagle, C. McMullen-Jackson, E. Meidan, E. Mellins, E. Mendoza, R. Mercado, A. Merritt, L. Michalowski, P. Miettunen, M. Miller, D. Milojevic, E. Mirizio, E. Misajon, M. Mitchell, R. Modica, S. Mohan, K. Moore, L. Moorthy, S. Morgan, E. Morgan Dewitt, C. Moss, T. Moussa, V. Mruk, A. Murphy, E. Muscal, R. Nadler, B. Nahal, K. Nanda, N. Nasah, L. Nassi, S. Nativ, M. Natter, J. Neely, B. Nelson, L. Newhall, L. Ng, J. Nicholas, R. Nicolai, P. Nigrovic, J. Nocton, B. Nolan, E. Oberle, B. Obispo, B. O’Brien, T. O’Brien, O. Okeke, M. Oliver, J. Olson, K. O’Neil, K. Onel, A. Orandi, M. Orlando, S. Osei-Onomah, R. Oz, E. Pagano, A. Paller, N. Pan, S. Panupattanapong, M. Pardeo, J. Paredes, A. Parsons, J. Patel, K. Pentakota, P. Pepmueller, T. Pfeiffer, K. Phillippi, D. Pires Marafon, K. Phillippi, L. Ponder, R. Pooni, S. Prahalad, S. Pratt, S. Protopapas, B. Puplava, J. Quach, M. Quinlan-Waters, C. Rabinovich, S. Radhakrishna, J. Rafko, J. Raisian, A. Rakestraw, C. Ramirez, E. Ramsay, S. Ramsey, R. Randell, A. Reed, A. Reed, A. Reed, H. Reid, K. Remmel, A. Repp, A. Reyes, A. Richmond, M. Riebschleger, S. Ringold, M. Riordan, M. Riskalla, M. Ritter, R. Rivas-Chacon, A. Robinson, E. Rodela, M. Rodriquez, K. Rojas, T. Ronis, M. Rosenkranz, B. Rosolowski, H. Rothermel, D. Rothman, E. Roth-Wojcicki, K. Rouster-Stevens, T. Rubinstein, N. Ruth, N. Saad, S. Sabbagh, E. Sacco, R. Sadun, C. Sandborg, A. Sanni, L. Santiago, A. Sarkissian, S. Savani, L. Scalzi, L. Schanberg, S. Scharnhorst, K. Schikler, A. Schlefman, H. Schmeling, K. Schmidt, E. Schmitt, R. Schneider, K. Schollaert-Fitch, G. Schulert, T. Seay, C. Seper, J. Shalen, R. Sheets, A. Shelly, S. Shenoi, K. Shergill, J. Shirley, M. Shishov, C. Shivers, E. Silverman, N. Singer, V. Sivaraman, J. Sletten, A. Smith, C. Smith, J. Smith, J. Smith, E. Smitherman, J. Soep, M. Son, S. Spence, L. Spiegel, J. Spitznagle, R. Sran, H. Srinivasalu, H. Stapp, K. Steigerwald, Y. Sterba Rakovchik, S. Stern, A. Stevens, B. Stevens, R. Stevenson, K. Stewart, C. Stingl, J. Stokes, M. Stoll, E. Stringer, S. Sule, J. Sumner, R. Sundel, M. Sutter, R. Syed, G. Syverson, A. Szymanski, S. Taber, R. Tal, A. Tambralli, A. Taneja, T. Tanner, S. Tapani, G. Tarshish, S. Tarvin, L. Tate, A. Taxter, J. Taylor, M. Terry, M. Tesher, A. Thatayatikom, B. Thomas, K. Tiffany, T. Ting, A. Tipp, D. Toib, K. Torok, C. Toruner, H. Tory, M. Toth, S. Tse, V. Tubwell, M. Twilt, S. Uriguen, T. Valcarcel, H. Van Mater, L. Vannoy, C. Varghese, N. Vasquez, K. Vazzana, R. Vehe, K. Veiga, J. Velez, J. Verbsky, G. Vilar, N. Volpe, E. von Scheven, S. Vora, J. Wagner, L. Wagner-Weiner, D. Wahezi, H. Waite, J. Walker, H. Walters, T. Wampler Muskardin, L. Waqar, M. Waterfield, M. Watson, A. Watts, P. Weiser, J. Weiss, P. Weiss, E. Wershba, A. White, C. Williams, A. Wise, J. Woo, L. Woolnough, T. Wright, E. Wu, A. Yalcindag, M. Yee, E. Yen, R. Yeung, K. Yomogida, Q. Yu, R. Zapata, A. Zartoshti, A. Zeft, R. Zeft, Y. Zhang, Y. Zhao, A. Zhu, C. Zic

**Affiliations:** 1 University of California, San Francisco, 550 16th Street, 4th Floor, Box #0632, San Francisco, CA 94158 USA; 2 Hospital for Special Surgery, New York, NY USA; 3 Weill Cornell Medicine, New York, NY USA

**Keywords:** Social determinants of health, Polyarticular juvenile idiopathic arthritis, Health disparities, Disease activity

## Abstract

**Background:**

Social determinants of health (SDH) greatly influence outcomes during the first year of treatment in rheumatoid arthritis, a disease similar to polyarticular juvenile idiopathic arthritis (pJIA). We investigated the correlation of community poverty level and other SDH with the persistence of moderate to severe disease activity and functional disability over the first year of treatment in pJIA patients enrolled in the Childhood Arthritis and Rheumatology Research Alliance Registry.

**Methods:**

In this cohort study, unadjusted and adjusted generalized linear mixed effects models analyzed the effect of community poverty and other SDH on disease activity, using the clinical Juvenile Arthritis Disease Activity Score-10, and disability, using the Child Health Assessment Questionnaire, measured at baseline, 6, and 12 months.

**Results:**

One thousand six hundred eighty-four patients were identified. High community poverty (≥20% living below the federal poverty level) was associated with increased odds of functional disability (OR 1.82, 95% CI 1.28-2.60) but was not statistically significant after adjustment (aOR 1.23, 95% CI 0.81-1.86) and was not associated with increased disease activity. Non-white race/ethnicity was associated with higher disease activity (aOR 2.48, 95% CI: 1.41-4.36). Lower self-reported household income was associated with higher disease activity and persistent functional disability. Public insurance (aOR 1.56, 95% CI 1.06-2.29) and low family education (aOR 1.89, 95% CI 1.14-3.12) was associated with persistent functional disability.

**Conclusion:**

High community poverty level was associated with persistent functional disability in unadjusted analysis but not with persistent moderate to high disease activity. Race/ethnicity and other SDH were associated with persistent disease activity and functional disability.

## Background

Polyarticular Juvenile Idiopathic Arthritis (pJIA) is a chronic inflammatory arthritis affecting five or more joints in the first 6 months of onset in children aged sixteen and younger. Children with pJIA may experience prolonged periods of active disease [1] with an increased risk for a relapsing course throughout their lifespan [2]. Previous studies show that up to 70% of children report disability and limitation of activities into adulthood [3–6]. In adult rheumatoid arthritis (RA), a disease similar to pJIA, persistent moderate disease activity during the first year of treatment is associated with greater radiographic progression at 3 years, increased functional disability, and a 10-fold less chance of achieving clinical remission long term [7].

A compelling body of public health research suggests that clinical care accounts for approximately 20% of health outcomes, while socioeconomic, behavioral, and environmental factors determine the remaining 80% [8–12]. These social determinants of health (SDH) encompass “the circumstances in which people are born, grow up, live, work, and age” [13] and have a significant impact on the health outcomes of many chronic diseases, including RA [14]. Among patients with RA, socioeconomic factors (SEF), such as low education, poverty, lack of health insurance or use of public health insurance, are associated with high disease activity and poor functional status at presentation to a rheumatologist [15–17] and have been associated with worse functional capacity, higher disease burden, and more swollen and tender joints over the first year of treatment and beyond in patients with RA [18–22]. Health outcomes have been shown to be associated with the socioeconomic environment of an individual’s neighborhood [23–25], independent of an individual’s SEF. Features of community deprivation, such as community poverty level, are associated with sparse community resources needed for optimal health [26] with poor health outcomes consistently reported in communities where ≥20% of the population lives below the poverty level [27–29]. Importantly, community level poverty extends beyond individual wealth and includes the communal characteristics of a geographic area including poverty and race as well as the proportion of single-parent households, degree of racial segregation, and overall residential instability [30].

Unfortunately, there is limited research in this very important aspect of health inequity in JIA. A prior UK cohort study of children with JIA using geographic deprivation indices to capture socioeconomic status (SES) did demonstrate higher functional disability among Child Health Assessment Questionnaire (CHAQ) scores in those of lowest SES despite similar disease activity scores [31]. The impact of community level social deprivation on disease activity and functional disability during the first year of pJIA treatment has overall not been well described. Understanding the relationship between this important SDH and disease outcomes during the first year of treatment in a North American cohort is critical to identifying the highest-risk JIA patients who may benefit from early aggressive therapy and other interventions.

In this study, we examined the correlation of SDH and SEF, including community level poverty, with disease activity and functional disability during the first year of treatment among pJIA patients enrolled in the Childhood Arthritis and Rheumatology Research Alliance (CARRA) Registry, a large cohort of pJIA patients from North America.

## Patients and methods

### Study design

We retrospectively analyzed baseline through 12-month data collected from patients with pJIA in the CARRA Registry [32]. The Registry includes data from children with rheumatic diseases obtained by convenience sample from 70 sites centered at tertiary or quaternary care hospital and clinics providing pediatric rheumatology care across North America with all but four of these centers located in the United States. Patients diagnosed with pJIA are enrolled into the Registry by a pediatric rheumatologist before the age of 21. Research personnel from each CARRA site enter pertinent demographic and clinical data from registered patients with pJIA during initial and subsequent follow-up patient visits. Data utilized for this study were collected from April 2015 through February 2020.

### Analytical sample

Inclusion criteria were U.S. residency with a valid 5-digit zip-code and reported pJIA diagnosis with onset of symptoms prior to age 16, ≥5 joints involved in the first 6 months of disease, and Registry enrollment near the time of diagnosis (between 1 to 6 months from onset of symptoms). Patients with an additional diagnosis of other systemic inflammatory or autoimmune disease were excluded. Approval for exemption was obtained from the Hospital for Special Surgery and University of California, San Francisco Institutional Review Boards.

### Primary exposure

The primary exposure was community poverty level at the time of enrollment in the CARRA Registry. Five-digit zip-codes of the addresses where patients were located at symptom onset were queried. We geocoded these individual zip-codes to link patients to specific census tracts using census tract crosswalk files. Census tract level socioeconomic variables were obtained from the 2015-2019 American Community Survey (ACS) data using the Geographic Information Systems [33]. These geographic units are designed to be homogeneous with respect to population characteristics, economic status, and living conditions. The percentage of individuals living below the poverty line at the census-tract level is highly sensitive to gradients in health [26] with poor health outcomes consistently reported in communities where ≥20% of the population live below the poverty level [19, 27]. Therefore, community poverty level as a primary exposure was dichotomized as a concentration less than 20% (low community poverty level) and greater than or equal to 20% (high community poverty level).

Of note, self-reported household income was included as a variable in the Registry. However, this variable demonstrated significant missingness (10% prefer not to answer and 17% missing) and therefore was only used for sensitivity analyses.

### Primary outcomes

The primary outcomes were disease activity, as measured by the Clinical Juvenile Arthritis Disease Activity Score-10 (cJADAS-10) [34], and functional disability, as measured by the Child Health Assessment Questionnaire (CHAQ) [35], at baseline, 6, and 12 months. The cJADAS-10 is a composite measure that includes the number of active joints as defined by joint pain and swelling or limitation in range of motion, patient/parent global assessment, and physician global assessment of disease activity for a maximum score of 30 points. To identify patients with significant disease activity, we used a cutoff of 2.5 to delineate between mild disease activity and moderate to high disease activity as the goal in treatment is to achieve remission or mild disease activity within the first year of diagnosis [36]. The CHAQ is comprised of a series of questions to assess functional disability related to their primary diagnosis. The CHAQ has a maximum score of 3.0. We used a cutoff of 0.25 to delineate between the presence or absence of functional disability as has been used in prior studies [37, 38].

### Covariates

Covariates included demographic information and clinical characteristics from the baseline Registry visit. Demographics included patient age, self-reported race and ethnicity, self-reported household income, insurance status, and highest level of family education. Clinical covariates included rheumatoid factor (RF) and cyclic citrullinated peptide (CCP) antibody status.

Race and ethnicity were self-reported and recorded in the Registry as White, Asian, Black/African American/African or Afro-Caribbean, Hispanic/Latino/Spanish origin, “Other” (including Middle Eastern/North African, Native, American Indian or Alaska Native, Native Hawaiian or other Pacific Islander, mixed race, and other), and prefer not to answer. Self-reported household income level was defined as <$25,000, $25,000-49,999, $50,000-99,9999, and $100,000+. Family education level was defined as high school or less, college (including 1- 4-year college, junior college, or technical school), and graduate school. Insurance status was categorized as public insurance (including Medicaid, a state specific children’s insurance plan, and military health care), private insurance, or other (including no insurance, more than one, non-US insurance, other, or missing).

### Statistical analysis

Descriptive statistics were generated to describe the study population using number (N) [%] and median (IQR). Alluvial diagrams were created to qualitatively assess the proportion of key demographic variables of interest, including community poverty level, guardian education level, and insurance status, within the study cohort at baseline and 12 months. To account for multiple time points of measurement of our outcome variables (cJADAS-10 and CHAQ) at baseline, 6 months, and 12 months after diagnosis of pJIA, repeated measures analyses were performed. Despite expected changes in disease activity and disability over the first year of diagnosis due to initiation of treatment, we incorporated these 3 visits into our linear mixed effect models to understand the overall effect of community poverty level on disease disability and activity over the first year from diagnosis.

To understand the effect of community poverty level on disease activity via cJADAS-10, two analyses were performed. A generalized linear mixed effects model was used to assess the association between community poverty level (< 20% or ≥ 20%) and dichotomized cJADAS-10 score (≤2.5 or > 2.5 to delineate between inactive to mild versus moderate to severe disease activity [36]). This model was then adjusted for age, insurance status, race/ethnicity, sex, family education level, and RF/CCP status. In addition, we used a linear mixed effects regression model to determine the change in mean cJADAS-10 score for each unit change in community poverty level. The same covariates were adjusted for in this analysis.

To understand the effect of community poverty level on disease disability via CHAQ, a generalized linear mixed effects model was used to assess the association between community poverty level (< 20% or ≥ 20%) and dichotomized CHAQ score (< 0.25 or ≥ 0.25 to delineate between the presence or absence of functional disability [37, 38]). This model was adjusted for age at baseline visit, insurance status, race/ethnicity, sex, family education level, and RF/CCP status.

In all models, random subject-level intercepts were used. Statistical significance was evaluated at the 0.05 alpha level, and 95 % confidence intervals were generated for all predictor estimates. As the CARRA Registry represents a convenience sample of patients whose data would be retrospectively analyzed, all study subjects meeting inclusion and exclusion criteria were included for analysis and power calculations were not performed. All analyses were performed in R (4.0.3) for Windows.

### Sensitivity analysis

As a sensitivity analysis, we repeated the above analyses using self-reported household income as the primary predictor instead of community poverty level to account for individual level socioeconomic factors that may influence our primary outcomes.

## Results

### Baseline characteristics

Our study sample included 1684 participants who met the inclusion and exclusion criteria, out of the 3637 pJIA participants in the CARRA Registry from April 2015 through February 2020 (Table 1). Those excluded did not fulfill ILAR classification criteria for pJIA (including joint count < 5 at baseline and 6-month visits or reclassification to an alternate arthritis diagnosis over the course of follow up, enthesitis-related arthritis, psoriatic arthritis, systemic JIA, or oligoarticular JIA), had non-US residency, age ≥ 16, no Registry visit at 12 months, or entered the Registry more than 6 months after onset of symptoms. 19% of participants were living in neighborhoods with high community poverty (defined as ≥20% of the population living below the federal poverty line). The cohort was predominantly White (74%). There was a higher percentage of patients of non-White race living in communities with high compared to low community poverty (36.4% vs. 21.3%). A majority of the cohort reported an income level of $50,000 or higher (53.3%) though 26.7% of the cohort chose not to disclose their income. There was a greater percentage of families reporting the lowest income level of <$25,000 (19% vs. 8.8%) when they lived in communities with high poverty level. Additionally, those with high community poverty level reported lower education level (23.1% high school or less vs. 13.7%) and higher use of public insurance (43.0% vs. 21.5%). Among our primary outcomes, missingness was higher among the cJADAS-10 (38% at baseline; 50% at 6 months; and 55% at 12 months) compared to the CHAQ (8% at baseline; 27% at 6 months; and 28% at 12 months) scores.

**Table 1:** Clinical and demographic characteristics of the polyarticular juvenile idiopathic arthritis (pJIA) cohort (*N* = 1684) in the CARRA Registry (April 2015-February 2020) by community poverty level.

Characteristic	Overall (N = 1684)	Community Poverty Level < 20 (***N*** = 1368)	Community Poverty Level ≥ 20 (***N*** = 316)
**Sex**			
Male	362 (21%)	292 (21%)	70 (22%)
Female	1322 (79%)	1076 (79%)	246 (78%)
**Age at diagnosis** (Median, IQR)	7 (3-11)	7 (3-11)	8 (3-12)
**Race/ethnicity**			
White	1246 (74%)	1048 (77%)	198 (63%)
Asian	53 (3%)	46 (3%)	7 (2%)
Black, African American, African or Afro-Caribbean	63 (4%)	39 (3%)	24 (8%)
Hispanic, Latino, or Spanish origin	167 (10%)	109 (8%)	58 (18%)
Other	128 (8%)	103 (7%)	27 (9%)
Prefer not to answer	25 (1%)	23 (2%)	2 (<1%)
**Self-Reported Income Level**			
< $25,000	150 (9%)	99 (7%)	51 (16%)
$25-49,999	188 (11%)	136 (10%)	52 (17%)
$50-99,999	414 (24%)	324 (24%)	90 (28%)
≥ $100,000	483 (29%)	430 (31%)	53 (17%)
Prefer not to answer or unknown	449 (27%)	379 (28%)	70 (22%)
**Highest Level of Family Education**			
High school or less	260 (15%)	187 (14%)	73 (23%)
College, junior college, or technical school	656 (40%)	546 (40%)	110 (35%)
Graduate school or higher	289 (17%)	250 (18%)	39 (12%)
Prefer not to answer or unknown	479 (28%)	385 (28%)	94 (30%)
**Insurance**			
Public	430 (23%)	294 (22%)	136 (43%)
Private	1123 (67%)	970 (71%)	153 (48%)
Other	131 (10%)	104 (7%)	27 (9%)
**RF+**			
Positive	309 (18%)	233 (17%)	76 (24%)
Negative	1204 (72%)	997 (73%)	207 (66%)
Not performed or missing	171 (10%)	138 (10%)	33 (10%)
**CCP+ positive**			
Positive	252 (15%)	187 (14%)	65 (21%)
Negative	786 (47%)	649 (47%)	137 (43%)
Not performed or missing	646 (38%)	532 (39%)	114 (36%)

### cJADAS-10

**Fig. 1:**
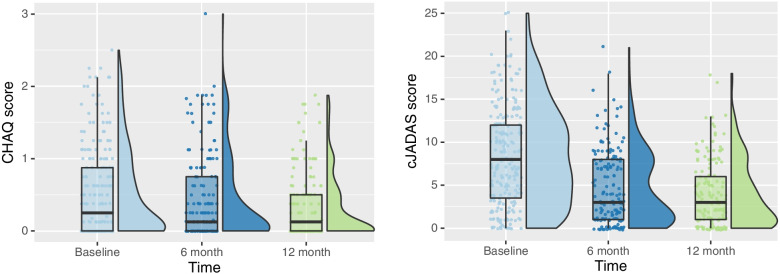
Box and violin plots demonstrate the distribution of the clinical Juvenile Arthritis Disease Activity Score-10 (cJADAS-10) (left) and Child Health Assessment Questionnaire (CHAQ) (right) scores among pJIA patients in the CARRA Registry at baseline, 6 months, and 12 months from time of diagnosis

**Fig. 2:**
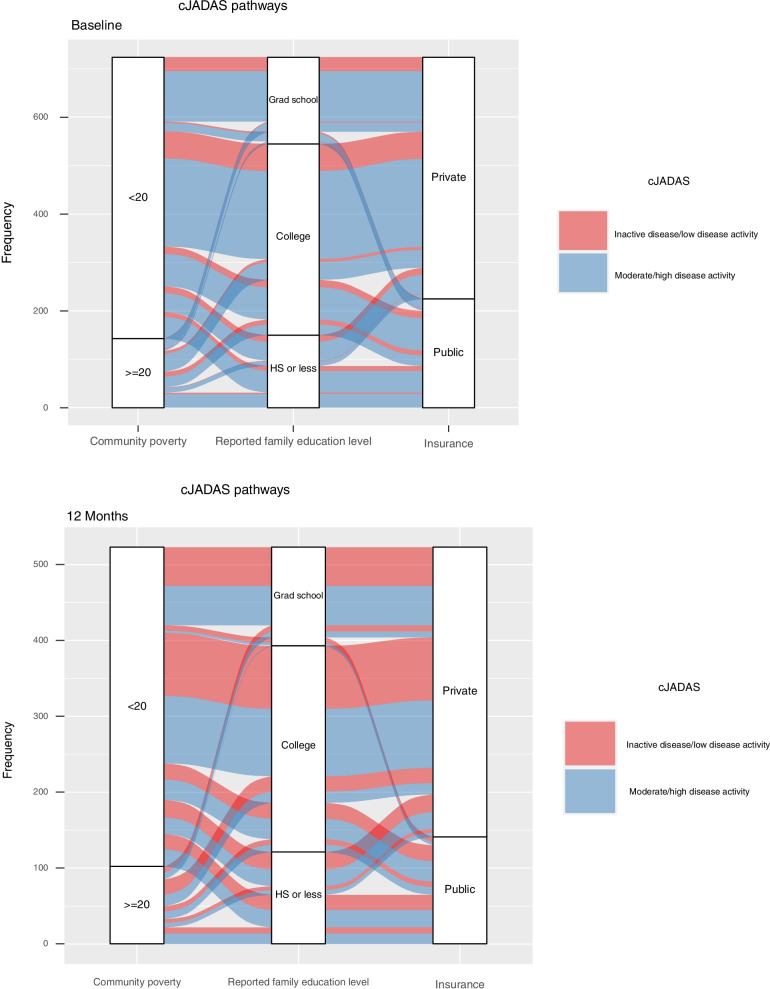
Alluvial diagrams demonstrate the flow of patient clusters by community poverty level, family education level, and insurance status on the Clinical Juvenile Arthritis Disease Activity Score-10 (cJADAS-10) among pJIA patients in the CARRA Registry at baseline (top) and 12 months (bottom)

Median cJADAS-10 scores from the pJIA cohort in the CARRA Registry decreased over the first year of therapy from baseline to 12 months (Fig. 1). Figure 2 is an alluvial diagram that represents the composition of key demographic features among the cohort at baseline and at 12 months. By visually following the red and blue “stream fields” between “blocks” of key demographic variables of interest, one can qualitatively assess differences in moderate to severe (blue) versus mild (red) disease severity among multiple demographic clusters of individuals at baseline (top) and 12 months (bottom). When comparing the baseline versus 12-month alluvial diagram, we can visualize trends in disease activity among these groups between baseline vs. 12 months from diagnosis. This figure highlights that a high proportion of patients had low disease activity or clinical remission at 12 months compared to baseline. However, those individuals with a high community poverty level (≥20%), family education level of high school or less, and with public insurance maintained the highest relative proportion of moderate to severe disease activity at 12 months post-diagnosis.

In our unadjusted generalized linear mixed effects model, those with high community poverty level did not have a statistically significant increase in odds of moderate to high disease activity over the first year of pJIA treatment (OR 1.14, 95% CI 0.85-1.53, *p*-value 0.4). This was also consistent after adjustment for age, sex, insurance status, race/ethnicity, family education level, and RF/CCP status (aOR 1.04, 95% CI 0.75-1.45, *p*-value 0.8).

In our adjusted linear mixed effects model analyzing cJADAS-10 as a continuous measure, those with high community poverty level had a mean cJADAS-10 0.33 points greater on average than those with low community poverty level, when adjusted for confounders, though this relationship was not statistically significant (95% CI − 0.40 to − 1.06, *p*-value 0.373). “Other” race/ethnicity yielded a 148% increase in the odds of moderate to severe disease activity compared to their White counterparts (OR 2.48 95% CI: 1.41-4.36, *p*-value 0.002) (Fig. 4A).

### CHAQ

**Fig. 3:**
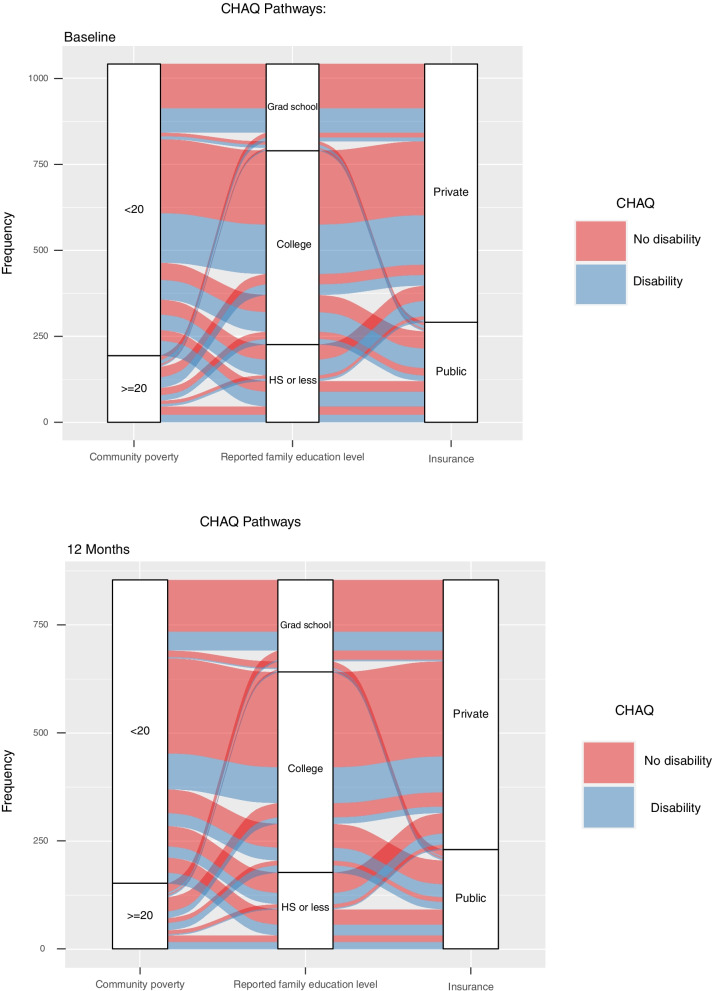
Alluvial diagrams demonstrate the flow of patient clusters by community poverty level, family education level, and insurance status on the Child Health Assessment Questionnaire (CHAQ) among pJIA patients in the CARRA Registry at baseline (A - top) and 12 months (B - bottom)

Figure 1 shows that median CHAQ scores improved over the first year of treatment among the pJIA cohort in the CARRA Registry. Figure 3 illustrates that the overall proportion of disability of patients with pJIA in the CARRA Registry appears to decrease at 12 months compared to the time of diagnosis. However, individuals with high community poverty level, low family education level (high school or less), and public insurance maintain the highest proportion of functional disability of all groups at 12 months post-diagnosis.

**Fig. 4:**
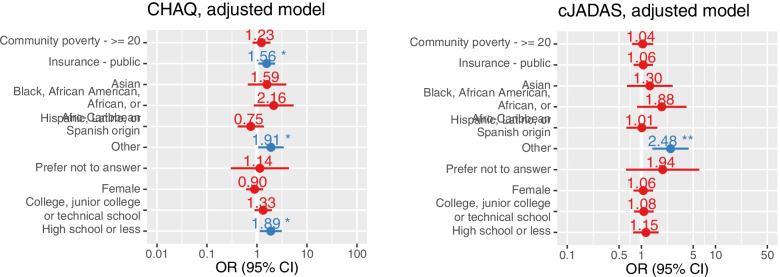
Adjusted generalized linear mixed effect models* analyzing the effect of community poverty level (< 20% versus ≥20%) on the odds of moderate to severe disease activity by cJADAS-10 score (**A** - left) and persistent functional disability by CHAQ score (**B** - right) among pJIA patients in the CARRA Registry. *Models designed with community poverty level as the primary predictor and adjusted for insurance status, race/ethnicity, family education level, age, sex, and RF/CCP status

In our unadjusted generalized linear mixed effects model, those with high community poverty level had an 82% increase in odds of having functional disability across the first year of treatment by CHAQ (OR 1.82, 95% CI 1.28-2.60, *p*-value < 0.001). On adjustment for age, sex, insurance status, race/ethnicity, family education level, and RF/CCP positivity, those with high community poverty level had a 23% increase in the odds of functional disability across the first year of treatment, though this association was no longer statistically significant (aOR 1.23, 95% CI 0.81-1.86, *p*-value 0.3). However, in the adjusted model, significantly increased odds of functional disability were associated with public insurance (aOR 1.56, 95% CI 1.06-2.29, *p*-value 0.023) as compared to private insurance, as well as “other” race/ethnicity (aOR 1.91, 95% CI 1.06-3.43, *p*-value 0.031) as compared to White, and high school or lower education level (aOR 1.89, 95% CI 1.14-3.12, *p*-value 0.013) as compared to those with graduate level education (Fig. 4B).

### Sensitivity analysis

Unadjusted and adjusted sensitivity analyses were performed using self-reported household income as the primary predictor to better understand the relationship between community level and individual level SDH and SEF on disease outcomes. We created generalized linear mixed effects models predicting disease activity by cJADAS-10 and disease disability by CHAQ (Table 2). In adjusted analysis, children from families earning $50,000-99,999 had 51% lower odds of moderate to severe disease activity compared to those from families earning <$25,000 per year (aOR 0.49, 95% CI: 0.28-0.85, *p*-value 0.011). Similarly, children from families making >$100,000 per year had lower odds of moderate to severe disease activity when compared to those families making <$25,000 per year (aOR 0.57, 95% CI 0.32-1.01, *p*-value 0.054). Similar trends were demonstrated in our analysis of disease-related disability. In adjusted analysis, those individuals from families making $50,000-99,999 per year had 65% lower odds of disease disability (OR 0.35, 95% CI 0.18-0.69, *p*-value 0.002) across the first year of diagnosis as compared to those making <$25,000, and those making >$100,000 per year had even lower odds of disability with an OR of 0.27 (95% CI: 0.13-0.55, *p*-value < 0.001); both relationships were statistically significant.

**Table 2:** Unadjusted and adjusted generalized linear mixed effect models^a^ analyzing the effect of self-reported income on the odds of moderate to severe disease activity by cJADAS-10 (top) and persistent disease disability by CHAQ (bottom) among pJIA patients in the CARRA Registry

	Unadjusted Model	95% CI	***p***-value	Adjusted Model	95% CI (*p*-value)	*p*-value
**cJADAS**						
< $25,000	–	–	–	–	–	–
$25-49,999	1.18	0.72-1.93	0.5	1.01	0.56-1.81	> 0.9
$50-99,999	0.65	0.43-0.99	**0.047**	0.49	0.28-0.85	**0.011**
≥ $100,000	0.64	0.42-0.96	**0.031**	0.57	0.32-1.01	0.054
**CHAQ**						
< $25,000	–	–	–	–	–	–
$25-49,999	0.65	0.36-1.17	0.2	0.58	0.29-1.17	0.13
$50-99,999	0.28	0.16-0.47	**< 0.001**	0.35	0.18-0.69	**0.002**
≥ $100,000	0.15	0.09-0.26	**< 0.001**	0.27	0.13-0.55	**< 0.001**

^a^Models adjusted for race/ethnicity, insurance status, family education level, age, sex, and RF/CCP status

## Discussion

Patients in the United States experience sizable health disparities rooted in social, economic, and environmental factors. For example, place of birth is more strongly associated with life expectancy than race or genetics [38] yielding a 15-year difference in life expectancy between the most advantaged and disadvantaged people in this country [38, 39]. These differences in health trajectory may be due to geographic characteristics, such as access to care and neighborhood safety, as well as health behaviors [13, 39] influenced by historical and social factors impacting the populations of people living in the United States. In this study of children with pJIA in the CARRA Registry, our primary aim was to investigate the association of community poverty level and other SDH with persistent moderate to severe disease activity and functional disability over the first year of diagnosis. Overall, our results do not show a statistically significant relationship between community poverty level and disease activity or disability, in adjusted analyses. However, individual-level poverty via self-reported income level, non-White race, public health insurance, and low guardian education level were all identified as statistically significant predictors of disease activity and disability in pJIA. Community and individual level SEF and SDH have separate and multiple pathways linking them to health [40]; these complexities appear to impact outcomes in pJIA.

In this study, we chose community poverty level as the primary predictor for outcomes in pJIA because the socioeconomic context of communities and neighborhoods affect the characteristics of the social, service, and physical environments to which all residents are exposed regardless of their own socioeconomic position [41, 42] and may have a greater negative impact on those with fewer individual resources [43, 44]. While community poverty level was not associated with an increase in odds of moderate-to-severe disease activity, those with high community poverty level did have higher disease activity scores (0.33 points greater on average than those with low community poverty level, in adjusted analysis). Prior work has identified that community poverty level was associated with a statistically significant delay in presentation to a pediatric rheumatologist [45]. These combined results may suggest that exposure to neighborhood level deprivation is more associated with delays in diagnosis or delays in care than in determining patient outcomes. Once care with pediatric rheumatology has been established, those children living in high areas of community poverty may not have increased risk for persistent disease activity or disability, which suggests some success in equitable care delivery for children with pJIA. It will be of critical importance to design interventions targeting more rapid referral to rheumatology and diagnosis of children with undiagnosed pJIA, possibly through increased education of childhood arthritis to primary care providers working in such areas, as well as targeting the underlying factors of neighborhood deprivation, including socioeconomic and racial inequalities.

Within our cohort at large, median scores for functional disability and disease activity decreased from the baseline visit to the 12-month visit across the cohort (Fig. 1), indicating that most pJIA patients in the CARRA Registry have improvement in functional disability and decreased disease activity with treatment of their arthritis. It is important to highlight that those children living in areas of high community poverty, lower guardian education level, and public insurance nearly universally presented with moderate-to-severe disease activity with the majority with persistent disease activity 12 months after enrollment (Fig. 2). This may suggest that exposure to multiple factors associated with social deprivation may accumulate and impact outcomes in pJIA, as has been shown in child health more generally [46] and should be an area of further research.

It is also possible that community poverty level may truly contribute to poor health outcomes for children with pJIA though its definition or measurement in our study was too broad. This measure is derived using 5-digit zip code data and thus lacks the specificity in geographic region captured by 9-digit zip code geocoded social indices, such as the Area Deprivation Index. Such measures could not be used as only 5-digit zip code data was available. Community poverty level lacks incorporation of other components of social deprivation at the community level, such as access to education and housing characteristics, in addition to income. It is possible that use of a 5-digit zip code-based method may inappropriately classify high-earning households as living in areas of high poverty (as evidenced by 17% of the high-community poverty level cohort reporting a household income level of >$100,000), leading to misclassification bias. As discussed previously, if community-level poverty inappropriately misclassifies individual-level poverty, the concept of community-level poverty includes characteristics of the physical location and environment to which all residents are exposed, regardless of socioeconomic position [41, 42]. It is also possible that an association that may have been present could have been missed due to selection bias relating to subjects who are enrolled in the CARRA Registry given low exposure to high community poverty level (only 19% of our cohort). In spite of these limitations, community poverty at the zip code level is widely used in public health and medical research.

Nevertheless, in adjusted models, several individual-level SDH were found to be associated with poor outcomes. The role that individual-level poverty level may play on outcomes is suggested by a possible protective effect of higher income (e.g., those patients with self-reported household income of ≥$50,000 had 65% lower odds of having moderate to high disease activity and had an average cJADAS-10 score at least 1.76 units lower compared to those with a household income of ≤$25,000 per year). Such findings suggest important relationships between individual poverty and disease activity in pJIA. Disparities in access to care are suggested by poorer outcomes among those with public insurance, including increased odds of persistent functional disability by 12 months. These findings are similar to a prior analysis of patients with JIA at a large academic pediatric rheumatology center where public insurance remained a statistically significant predictor of functional disability [47]. This could reflect inadequate coverage of medications or other important mitigators of disability, including physical and occupational therapy, between public versus private insurance leading to differential outcomes. It is possible that disparities in public insurance may depend on state of residence, as programs differ.

Finally, the role of education and health literacy may also be suggested by poorer outcomes experienced by those with lower family education level, including an 89% increase in odds of functional disability compared to families with graduate school education. The interplay between community-level and individual-level SDH contributing to disparities warrants further study. Improved understanding of which factors are related to delays in diagnosis and establishing care (such as community poverty level) versus those related to worse outcomes (such as insurance status) will be critical to understand in designing interventions to mitigate disparities.

Importantly, the patients included in this study are predominantly White and privately insured. Our findings do highlight that the CARRA Registry has become more diverse over time compared to prior iterations [48]. Prior research has shown that White patients generally fare better than non-White patients in mortality and morbidity status for many chronic illnesses [49, 50]. It is also recognized that Black and Latinx patients experience substantial health disparities compared to Whites [51]. Similar trends were also demonstrated in our cohort where Black patients had higher disease activity (on average cJADAS-10 score 1.73 times higher than White patients). Additionally, Non-White (including Middle Eastern/North African, Native, American Indian or Alaska Native, Native Hawaiian or other Pacific Islander, mixed race, and other) race/ethnicity was associated with persistent functional disability and higher disease activity over the first year from diagnosis. While not the primary focus of our work, our findings suggest the important role of race and ethnicity on disease activity and disability in pJIA, reflecting the larger problem that systemic racism poses to the health of our patients. These findings may have been attenuated by the racial and ethnic homogeneity of this cohort, which highlights the need to prioritize racially, ethnically, and geographically diverse patient enrollment to pediatric registries to understand the relationship more accurately between SDH and disease outcomes.

### Strengths

Strengths of this study include a large sample of patients from the CARRA Registry not limited to a single center or geographic location. An additional advantage was our cohort study design, which enabled longitudinal measurement of disease activity and functional disability. The repeated measures design of this study adjusts for within-subject variability and increases power by increasing the number of total observations for each participant, thus allowing us to identify important statistically significant differences between groups. Using both poverty level and self-reported household income, education, and insurance allows for a more nuanced assessment of the complicated relationship between community and individual SDH factors.

While this CARRA cohort may not be demographically representative of the overall pJIA population, the large sample size and repeated measures design has given us the opportunity to study the effect of community poverty as a potentially modifiable key step in the long-term care of these patients. Furthermore, we expect that the lack of diversity would bias our study towards the null and therefore does not threaten the validity of our findings.

### Limitations

We encountered missingness in our dataset for variables such as household education level (28.4%) and predictors such as reported income level (26.7%). Due to the reluctance of lower income earners to report financial details, this was likely a nonrandom nonresponse [52]. Our outcome measures, including the cJADAS-10, also had high degrees of missingness (i.e., 38% at baseline). Therefore, this may not reflect the true underlying relationship between SDH and income level with disease activity and functional disability during the first year of treatment. We considered but did not perform statistical imputation as the cJADAS-10 was calculated individually for each patient visit; with multiple components requiring imputation, this may have led to a biased estimate. Finally, this analysis does not provide information on the role that SDH may play in access to medications and other therapies, which will be the subject of future work.

### Future areas of study

While community poverty level in this analysis was not found to be a statistically significant predictor of disease activity or disability, future research should study other variables related to social disadvantage, such as the area deprivation index, as socioeconomic deprivation at the community level is still hypothesized to impact pJIA outcomes. Future studies should also examine the role of cumulative social disadvantage on disease outcomes in JIA, as well as the interrelationships between social determinants of health and race/ethnicity in creating racial and other health disparities in pJIA.

## Conclusions

Our study found evidence of socioeconomic factors and other SDH as predictors of moderate to high disease activity and persistent functional disability after the first year of treatment of pJIA. Although not statistically significant, we found a linear relationship between community poverty level and cJADAS-10 score. We uncovered racial and socioeconomic disparities in functional disability and disease activity outcomes affecting children with pJIA enrolled in the CARRA Registry. Identification of specific modifiable factors that influence disease-specific outcomes during this critical first year of treatment is needed, with the goal of reducing health disparities and promoting health equity across the population.

## Data Availability

The data that support the findings of this study are available from the CARRA Registry, but restrictions apply to the availability of these data, which were used under license for the current study, and so are not publicly available. Data are however available from the authors upon reasonable request and with permission of the CARRA Registry.

## Abbreviations

SDH: Social determinants of health
pJIA: Polyarticular juvenile idiopathic arthritis
SEF: Socioeconomic factors
CARRA: Childhood Arthritis and Rheumatology Research Alliance
ACS: American Community Survey
cJADAS-10: Clinical Juvenile Arthritis Disease Activity Score-10
CHAQ: Child Health Assessment Questionnaire
RF: Rheumatoid factor
CCP: Cyclic citrullinated peptide
